# COVID-19 Vaccine Hesitancy Among Black Women in the US

**DOI:** 10.1001/jamanetworkopen.2024.53511

**Published:** 2025-01-09

**Authors:** Brittany C. Slatton, Farrah G. Cambrice, Serwaa S. Omowale

**Affiliations:** 1 Department of Sociology, College of Liberal Arts and Behavioral Sciences, Texas Southern University, Houston; 2 Department of Sociology, Division of Social Sciences, Prairie View A and M University, Prairie View, Texas; 3 Department of Management, Policy, and Community Health, School of Public Health, The University of Texas Health Science Center, Houston

## Abstract

**Importance:**

COVID-19 vaccine hesitancy among Black women is a critical public health concern, potentially exacerbating existing health disparities and impacting community-wide vaccination efforts.

**Objective:**

To explore the factors associated with COVID-19 vaccine hesitancy among Black women in the US and identify the specific concerns and experiences shaping hesitant attitudes toward vaccination.

**Design, Setting, and Participants:**

Qualitative study using in-depth, semistructured interviews conducted virtually between June and November 2021. Thematic analysis was used to identify key themes from the interview data. Participants were recruited through social media platforms and word-of-mouth referrals from various regions of the US. Interviews were conducted remotely via Zoom. Data were analyzed from June to October 2023.

**Main Outcomes and Measures:**

Identification of key themes associated with COVID-19 vaccine hesitancy, identified through thematic analysis of interview data.

**Results:**

Fifty-four Black women aged 21 to 66 years participated in the study. Most participants (41 [75.9%]) resided in the South. The sample was predominantly well-educated, with 41 (75.9%) holding a bachelor’s degree or higher. Income levels varied, with 16 (29.6%) earning between $40 000 and $59 999 annually. Regarding family structure, 32 participants (59.3%) reported having no children, while 22 (40.7%) had 1 or more children. Three primary themes emerged as being associated with vaccine hesitancy: (1) mistrust in health care and government, rooted in past medical exploitation; (2) concerns over vaccine safety and long-term effects, particularly regarding reproductive health; and (3) ineffective and coercive vaccine communication and promotion. Participants expressed deep-rooted skepticism about being treated as “expendable” in medical research, fears about the rapid vaccine development process, and frustration with coercive vaccine advertisements. Many women desired more comprehensive education about vaccine mechanisms and criticized the use of coercive financial incentives and celebrity endorsements in vaccine promotion.

**Conclusions and Relevance:**

Addressing COVID-19 vaccine hesitancy among Black women requires a multifaceted approach that acknowledges historical traumas, provides clear and transparent safety information, and avoids coercive vaccine promotion strategies. These findings emphasize the need for health care practitioners and public health officials to prioritize trust-building, engage community leaders, and tailor interventions to address the unique concerns of Black women to improve vaccine confidence and uptake.

## Key Points

**Question:**

What factors are associated with COVID-19 vaccine hesitancy among Black women in the US?

**Findings:**

In this qualitative study of 54 Black women, 3 main themes emerged as being associated with vaccine hesitancy: mistrust in health care and government, concerns over vaccine safety and long-term effects, and ineffective and coercive vaccine communication and promotion. Participants expressed deep-rooted skepticism stemming from past medical exploitation, fears about rapid vaccine development, and frustration with inadequate and coercive vaccine messaging.

**Meaning:**

These findings suggest that addressing vaccine hesitancy among Black women requires acknowledging historical traumas, providing transparent safety information, and developing culturally respectful promotion strategies that avoid coercive tactics to build trust and improve vaccine uptake.

## Teaser

This qualitative study evaluates concerns and experiences associated with COVID-19 vaccine hesitancy among Black women in the US.

## Introduction

The COVID-19 pandemic has highlighted and intensified existing health disparities in the US, with Black communities experiencing disproportionately higher rates of infection and mortality.^1,2,3,4^ This disparity is rooted in structural racism—racism embedded in discriminatory policies and practices that create and perpetuate unequal access and outcomes^5^—along with social determinants of health, community density, and common underlying medical conditions.^6,7,8^ Black US residents’ COVID-19 vulnerability is also attributed to weathering—cumulative physiological wear-and-tear caused by sustained social, economic, and political adversity.^9^ This phenomenon weakens immune systems and organs, increasing infection susceptibility.^10^ Related is the concept of allostatic load, referring to cumulative physiological burden from chronic stress and repeated adaptation to stressors.^11,12,13^ Black women often experience higher allostatic load scores than Black men and White men and women,^10^ likely due to increased exposure to high-stress situations requiring elevated coping efforts.

While vaccine availability has improved since 2021, vaccine hesitancy remains a persistent challenge.^14,15^ Defined as “the delay in acceptance or refusal of vaccines despite their availability,”^16,17^ hesitancy is a complex phenomenon influenced by emotional, cultural, social, spiritual, and political factors.^18^ It exists on a continuum from outright refusal to doubts about specific vaccines.^19^ The evolving nature of COVID-19 vaccine hesitancy is framed by political polarization, social media influence, and concerns about long-term effects.^18,20,21,22^

Black US residents’ show higher levels of vaccine hesitancy than their White counterparts.^23,24,25,26^ Their hesitancy often stems from medical mistrust—distrust of health care systems, practitioners, and treatments^27,28^ rooted in historical medical mistreatment and structural racism^29,30^—as well as lack of trust in government and health care institutions, concerns about rapid vaccine development, and lack of diversity in clinical trials.^31,32,33,34,35,36,37^ Black women appear to be more hesitant than Black men, with 19% of Black women in 2021 expressing definite refusal to receive the COVID-19 vaccine, a percentage double that of Black men.^38^ As primary family health care decision-makers, Black women’s vaccine hesitancy can influence family members’ decisions and reduce overall vaccine uptake.^38,39^ It is important to note that Black women are not a homogeneous group, and certain subgroups of Black women, such as pregnant women, may be more vulnerable to vaccine hesitancy. While vaccine hesitancy patterns have evolved since 2021, understanding the root causes and ongoing dynamics remains crucial. The current literature on vaccine hesitancy among Black women is limited, with many studies conducted before vaccine availability, focusing on broader racial and/or ethnic populations or limited by sample size.^24,25,36^ Hence, this qualitative study was developed to undertake a more nuanced examination of the specific dynamics influencing COVID-19 vaccine hesitancy among Black women in the US. By analyzing data from 2021, a critical period in vaccine rollout, this study provides insights into the formative stages of hesitancy that continue to shape current attitudes and behaviors.^14,15^ Gaining insight into vaccine hesitancy among Black women is crucial for developing effective public health strategies that address health disparities and improve vaccine uptake.

## Methods

### Study Design, Participants, and Data Collection

This qualitative study was approved by Texas Southern University’s institutional review board and followed Standards for Reporting Qualitative Research (SRQR) reporting guidelines. All participants provided written and verbal informed consent before their involvement. The study used in-depth interviews to explore Black women’s perspectives, facilitating a rich and nuanced understanding of the participants’ lived experiences.^40^

Eligibility criteria for participation included being a Black woman, age 18 and over, and living in the US. Participants self-selected for this study and self-identified their race as Black or African American. The first author (B.C.S.) recruited Black women through purposive sampling via social media platforms, including LinkedIn and via word-of-mouth referrals. She conducted semistructured interviews virtually through Zoom between June and November 2021. Each interview lasted approximately 75 minutes and was audio-recorded with the participants’ consent. Participants received a $25 incentive in appreciation of their time and contributions. Participants were asked open-ended questions about COVID-19 and vaccination. Follow-up questions were employed to encourage participants to explain their concerns or provide specific examples. Examples of questions asked include: “What are your thoughts on the COVID-19 vaccine?,” “What factors influence your decision to get vaccinated or not?,” and “What COVID-19 vaccine advertisements or messages have you seen?”

To foster trust and cultural connections, the research team of 3 Black women operated from a social justice perspective, which values participants’ contextually relevant insights and validates their role as knowledge producers.^40,41,42^ Strategies employed to create and maintain trust among participants included recognizing the researchers’ positionality by engaging in reflexivity, which means being mindful of how their identities as Black women could influence the data collection process.^43^ To build and sustain trust with participants, the researchers focused on fostering cultural connections and remained open and nonjudgmental toward different perspectives.^44,45,46,47,48,49^

### Data Management

Audio recordings were transcribed using a paid automated transcription service accessible only on a password-protected device. Participants’ identifying information was removed from transcripts to maintain anonymity and transcripts were reviewed and corrected for any errors. The service employs robust security measures, including internal access controls and encryption. The first author stored all study data, including audio recordings, transcripts, and participant information, on a password-protected computer in a locked home office to ensure data protection. Access to the data was limited to research team members directly involved in the study.

### Data Analysis

The first author and an external reviewer independently analyzed the data using the Braun and Clarke^50^ 6-step thematic analysis approach, following an inductive method that allowed themes to emerge organically from the data, without preconceived categories. This process involved iterative coding, constant comparison, and collaborative refinement of themes, as detailed in the Figure. The first author and the external reviewer read and reread the interview transcripts to become familiar with the data. They then independently coded the data, highlighting relevant words, phrases, or passages. The external reviewer used NVivo 14 qualitative software (Lumivero) to assist with organizing and analyzing the data. After coding, the first author and external reviewer compared their codes, discussing any discrepancies until reaching consensus. The codes were then collated into potential themes and subthemes, which were repeatedly reviewed and refined to accurately represent the dataset. For clarity and consistency, the second and third authors (F.G.C. and S.S.O.) reviewed the resulting themes and representative quotes. Data were analyzed from June to October 2023.

**Figure.**
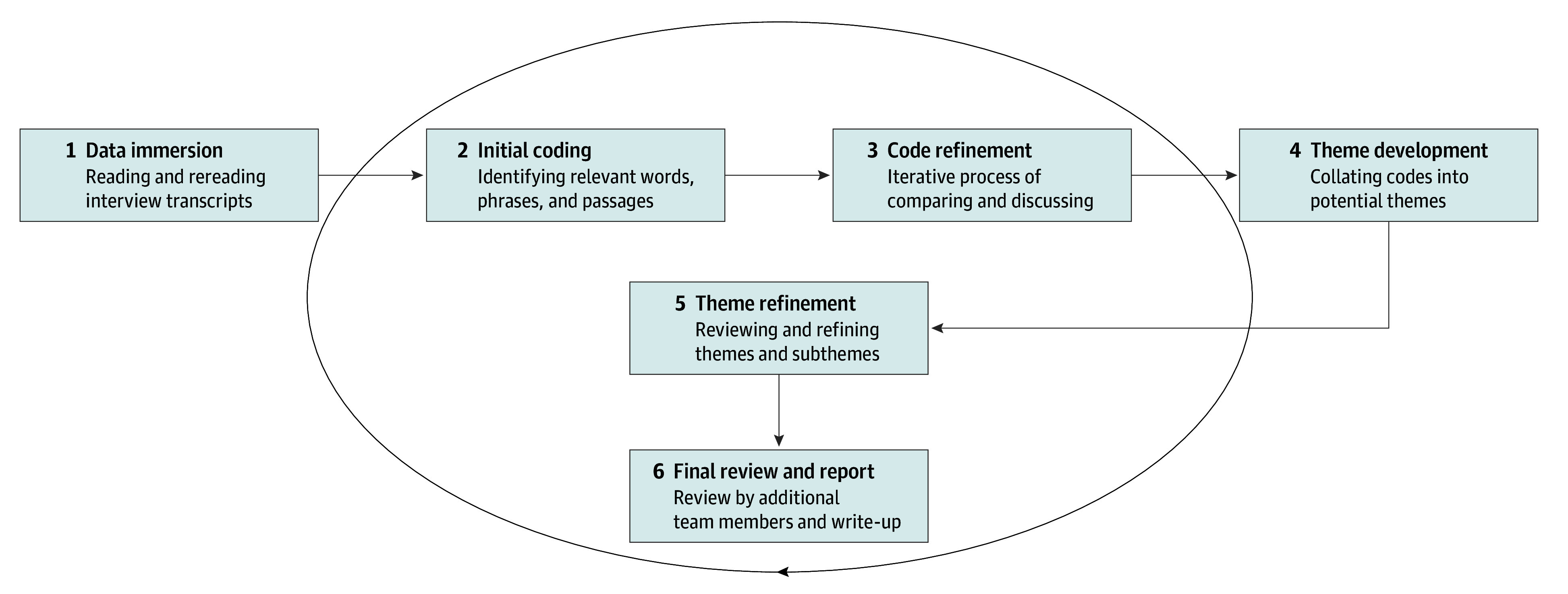
Six-Step Inductive Thematic Analysis Process This diagram illustrates the iterative, inductive process of thematic analysis used in our study, derived from Braun and Clarke’s methodology.^50^ The process progresses through 6 interconnected steps: data immersion, initial coding, code refinement, theme development, theme refinement, and final review and report. Each step allows for continuous refinement and analysis of the data, ensuring a thorough examination of the qualitative data collected from interviews. The circular arrow represents the iterative nature of the process, enabling researchers to move between steps as needed, deepening their understanding and refining their analysis throughout the study.

## Results

Fifty-four Black women comprised the study sample; Table 1 details their demographics. Participants ranged in age from 21 to 66 years. The majority of participants were highly educated, with 41 (75.9%) holding a bachelor’s degree or higher. Income levels varied, with the largest group (16 participants [29.6%]) earning between $40 000 and $59 999 annually, while 8 participants (14.8%) reported incomes of $80 000 or more. Regarding family structure, 32 participants (59.3%) reported having no children, while 22 (40.7%) had 1 or more children. The sample was geographically concentrated in the South, with 41 participants (75.9%) residing in this region. A significant majority (39 participants [72.2%]) identified as Christian, with the remainder distributed among various beliefs or nonbeliefs.

**Table 1.** Sample Demographics

Characteristics	Participants, No. (%) (n = 54)
Age, range, y	21-66
Education	
High school	3 (5.55)
Some college	6 (11.11)
Associate’s degree	4 (7.41)
Bachelor’s degree	15 (27.78)
Master’s degree	22 (40.74)
PhD or professional degree	4 (7.41)
Annual income, US $	
0-19 999	7 (12.96)
20 000-39 999	13 (24.07)
40 000-59 999	16 (29.63)
60 000-79 999	10 (18.52)
80 000-99 999	4 (7.41)
≥100 000	4 (7.41)
Children	
None	32 (59.26)
≥1	22 (40.74)
Region	
Midwest	9 (16.67)
South	41 (75.93)
West	2 (3.70)
Northeast	2 (3.70)
Religion	
Christian	39 (72.22)
Agnostic	4 (7.41)
Buddhist	1 (1.85)
Atheist	1 (1.85)
Other	9 (16.67)

The in-depth interviews revealed 3 significant themes associated with COVID-19 vaccine hesitancy within this population. Table 2 presents an overview of the themes along with representative quotes from the participants.

**Table 2.** Themes and Representative Quotes

Themes	Representative quotes
Mistrust in health care and government	“[The vaccine] actually a complete turn off to me because of the history of vaccinations in the African American community. These were basically used as poison for us. So, which is actually why I moved forward in a holistic manner most of the time, because I do not wanna put that type of chemical and everything in my body. I don’t know how it’s going to interact with me. That’s why I’m antivaccination. Um, but I, I never like the fact that we are always the, uh, uh, the lab experiment, the testing community for these vaccinations. Why are we always so, uh, expendable for your research?”
	“it happened way too fast. Yeah. Um, you know, and in, in comparison to some of the other things that they’ve [government] been trying to find cures for vaccines for, they haven’t been able to, but they got this. But then, you know, a year’s time-ish, like no, that just doesn’t sound right.”
	“Honestly because I trust my mother’s opinion more than I trust the government and the CDC. And it’s her job [she’s a pharmacist] to know how long medication has been in the works, right?”
	“I’m sitting there like, y’all understand what airborne means, right? We have 2 negative pressure rooms in every hospital that is for airborne. So where y’all gonna put the rest of these people that are infected with an airborne disease? The CDC must have thought about that cause the next time I came to my shift, which was 12 hours later, all of a sudden it’s [COVID] droplet now.”
	“I just do not like the fact that they [vaccines] are targeted to the Black community to serve us as lab experiments.”
Concerns over vaccine safety and long-term effects	“I was definitely nervous about all of the, uh, nervous about what could happen immediately following, I was nervous about possibly having a bad reaction.”
	“I’m nervous to see like if there, like how the research will be like 10 years from now.”
	“When I got the vaccine, um, the girl, the physician, the pharmacist was saying to me, um, you know, that it would affect your cycle. Yeah. But they just figured that out so not many people know about it.”
	“My mom’s thing is I’m not gonna do anything to provoke it. Especially because they said these…some of these vaccines are creating, are causing blood clots.”
	“And so with the vaccine, I don’t have faith that if I have an allergic reaction, they’re gonna tie into, oh, it’s the vaccine. They’re gonna try to push it off as something else.”
	“It was just more like, um, scientifically I hadn’t seen what the results were. Like I saw people like getting tested for it, but I wasn’t seeing like were there any permanent long-term effects.”
	“….And all these vaccines came out way too soon. And that’s why all these people are having these side effects. That’s why the people who are doing the Johnson and Johnson are getting the blood clots. And now they’re coming out with booster shots and these shots have never been out. How effective actually are these shots?”
	“Just looking at how it’s affected other people. My body chemistry is not made up like everybody else’s, you know? This could affect me negatively.”
Ineffective and coercive vaccine communication and promotion	“I think it started off, um, I think the advertisement started off pretty straightforward and um, they were not providing enough like educational background on the need for why we needed to get the Covid vaccine.”
	“I mean people just don’t, no one has explained how a vaccine works, to be like, oh it’s new. And it’s like, well this part’s new, but this is why it’s not like a permanent life. Like this is why it’s not a permanent thing. This is why it’s considered safe.”
	“It’s been mostly minorities and so there is a distrust and so you have to go back to the messaging. When you roll out a project, you have to make sure that your messaging is on point.”
	“Well, I just felt like there was this major push, um, especially in our communities, you know, like even when I saw, um, what is his name back that, back, that thing up…Juvenile [a famous rapper]. He did Juvenile. Yeah. Juvenile did an ad and it was like, it was called ‘vax, that thing up’ like [the song] ‘back, that thing up.’ It was ‘vax that thing up.’ So when I saw that, I was like, oh my God. Like they are really trying to appeal to the African American community, right? Yeah. And so I was like, okay, this is crazy. And then of course, like paying people to get the vaccine and, you know, just all of that. And I was just like, okay, I get it. Like, you know, but it was just, it was just kind of weird to me of how they were just like really shoving it down our throats, you know?”
	“I mean, I don’t really watch a lot of TV, but I would see stuff like on, you know, social media. They would make kind of jokes about some of the advertisements. They look like they were like racially insensitive.”
	“Horrible. I think it’s horrible. I think it’s horrible. I just had a paper on my door talking about you could win a million dollars in the lottery if you get this COVID vaccine. I live in [city] and I live on the east side and there is poor Black people in this area. Okay, you know, everybody’s gonna sign up for that. Everyone’s gonna sign up. I think it’s sick. I think it’s sick.”
	“Because that’s definitely something I noticed as well. And I think that that happens often when it comes to the black community, the, the emphasis on, well, this celebrity is doing X, Y, and Z and so because a celebrity is doing it, you should do it too. As if we don’t wanna know actual numbers and facts and statistics, facts.”

### Theme 1: Mistrust in Health Care and Government

A prominent theme that emerged from the interviews was the deep-rooted mistrust in the health care system and government, which was associated with Black women’s hesitancy toward the COVID-19 vaccine. Participants expressed concerns about being treated as “expendable” and “lab experiments” in the context of vaccine development. Participant 37 shared, “So um [vaccines] actually a complete turn off to me is because of the history of vaccinations in the African American community, these were basically used as poison for us.” She added, “That’s why I’m antivaccination…I never like the fact that we [African Americans] are always the…lab experiment, the testing community for these vaccinations.” This sentiment echoes historical instances of medical exploitation, which participants mentioned as influencing their current views.

Present day mistrust appeared to be further exacerbated by rapidly changing official communications during the pandemic, as illustrated by a health care professional’s account. This participant stated, “…We have 2 negative pressure rooms in every hospital that is for airborne. So where y’all gonna put the rest of these people that are infected with an airborne disease? The CDC must have thought about that cause the next time I came to my shift, which was 12 hours later, all of a sudden it’s [COVID] droplet now” (participant 53). This account illustrates how the health care professional’s preexisting skepticism of the government was further exacerbated by rapidly changing official guidelines, which intensified her decision against vaccination.

Participants also expressed skepticism toward the government’s real motives, questioning how they could rapidly develop a COVID-19 vaccine while failing to cure longstanding diseases. Participant 17 noted, “It happened way too fast... in comparison to some of the other things that they’ve [government] been trying to find cures for... they haven’t been able to, but they got this?”

### Theme 2: Concerns Over Vaccine Safety and Long-Term Effects

Participants expressed apprehension about immediate adverse effects and long-term impacts of the COVID-19 vaccine on their health. Participant 6 voiced immediate concerns: “I was definitely nervous about... what could happen immediately following, I was nervous about possibly having a bad reaction.” Some participants doubted health care professionals’ ability to accurately attribute adverse reactions to the vaccine. Participant 38 elaborated, “And so with the vaccine, I don’t have faith that if I have an allergic reaction, they’re gonna tie into, oh, it’s the vaccine. They’re gonna try to push it off as something else.” This concern stemmed from this participant’s earlier experience of having allergic reactions from other health issues dismissed by doctors, reflecting a deeper mistrust in the health care system’s responsiveness to her health concerns.

Long-term consequences were a major worry, particularly due to the vaccine’s novelty. Participant 6 expressed, “I’m nervous to see... how the research will be like 10 years from now.” Participant 34 added, “It was just more like, um, scientifically I hadn’t seen what the results were... I wasn’t seeing like were there any permanent long-term effects.” The rapid development of the vaccine intensified these concerns.

The perception of insufficient FDA approval was further associated with safety concerns. Participant 30 stated, “It’s not FDA approved...” This participant’s view stemmed from conversations she had with health care professionals who, in her view, did not adequately answer her questions about the vaccine. Rooted in this experience and past negative reactions with the flu vaccine, she questioned the safety of the COVID-19 vaccine, saying it “needed more time.”

### Theme 3: Ineffective and Coercive Vaccine Communication and Promotion

Participants expressed concerns about COVID-19 vaccine communication and promotion strategies. Participants felt that the information provided was unclear, inconsistent, and insufficient, leading to confusion and mistrust. They also perceived certain promotional tactics as unethically coercive, culturally insensitive, and disrespectful of their communities’ needs, particularly criticizing the use of financial incentives and celebrity advertisements.

Participants highlighted the need for more comprehensive education about vaccine mechanisms. One participant noted, “No one has explained how a vaccine works... this is why it’s not permanent, this is why it’s considered safe” (participant 14). The absence of clear explanations left many with unanswered questions and concerns. Inconsistency in vaccine advertisements was also problematic. Participant 18 observed, “Some [ads] are more informative, some of them are more meant to be like more persuasive.” This lack of uniformity in messaging appeared to erode trust in vaccine communication, with several participants expressing confusion about which sources to trust. As Participant 24 noted, “We heard so many conspiracy theories and didn’t know what to think,” highlighting the challenge of navigating conflicting information.

Participant 53 expressed disgust with lottery-style incentives, stating, “I just had a paper on my door talking about you could win a million dollars in the lottery if you get this COVID vaccine... I live on the east side and there is poor Black people in this area. Okay, you know, everybody’s gonna sign up for that …” This response underscores the perceived exploitation of vulnerable communities through financial incentives. It suggests that such promotion tactics may be viewed as manipulative, eroding trust rather than building it.

Culturally insensitive celebrity advertisements were particularly alienating. One participant described an ad featuring rapper Juvenile, saying, “Juvenile did an ad and it was like, it was called ‘vax, that thing up’ like [the song] ‘back, that thing up’…they are really trying to appeal to the African American community... they were just like really shoving it down our throats” (participant 11). Participants viewed celebrity-driven ads such as this as disrespectful to Black people’s intelligence, expressing frustration at the assumption that they would simply follow celebrities’ leads rather than make informed decisions based on real data. Participant 21 lamented, “As if we don’t wanna know actual numbers and facts and statistics …” highlighting the desire for factual information over celebrity coercion.

## Discussion

This qualitative study supports and extends existing research on vaccine hesitancy among Black women. Our findings align with earlier studies that find mistrust in health care systems and government as a significant factor potentially contributing to vaccine hesitancy. This mistrust is rooted in Black experiences of historical medical exploitation and unethical practices, as exemplified by the Tuskegee Syphilis Study and the case of Henrietta Lacks.^31,36,51,52^ Participants also expressed apprehension about immediate adverse effects and long-term impacts and doubted health care professionals’ ability to accurately diagnose and address vaccine-related reactions, reflecting broader issues of trust in medical care. The rapid vaccine development and deployment intensified concerns about vaccine safety, prompting questions about research adequacy and testing. The study found significant shortcomings in vaccine communication and expands the literature on how coercive vaccine promotion advertisements may shape hesitancy among Black women. Participants were particularly concerned about the use of advertisements that employed exploitative financial incentives and culturally disrespectful assumptions. They desired clear explanations about the vaccine’s mechanism of action, safety profile, and the rationale behind its importance.

This study further extends the existing literature by providing a nuanced exploration of COVID-19 vaccine hesitancy specifically among Black women, a group often overlooked in broader studies. By focusing on Black women’s experiences, this research offers insights into how the intersection of race and gender shapes vaccine attitudes and health decisions. The experiences of Black women are not simply the sum of racial and gender identities, but a unique form of experience that emerges from the interaction of these identities.^53^ Numerous historical and contemporary accounts of the mistreatment and exploitation of Black women in medical research and health care practices warrant studies that focus specifically on this group.^54^ Black women’s perspectives and lived experiences shaped by their social status^46,53^ are crucial for understanding their health behaviors and decisions. Our findings suggest that Black women’s vaccine hesitancy is influenced by their intersectional experiences of both racial and gender discrimination in health care settings. For example, participant 38’s experience of health care practitioners dismissing her health concerns reflects the challenges Black women face in the health care system,^55^ which may inform their vaccine decisions.

This study’s results have important implications for public health practice and policy. Health care practitioners and public health professionals should prioritize building trust and rapport with Black women, recognizing their pivotal role in health care decision-making within families and communities.^38,39^ Building trust may require training health care practitioners to be more culturally competent or skilled in understanding variations in Black women’s beliefs, norms, and history of discrimination. Previously published research on the flu vaccine suggests a positive association between access to culturally competent care and vaccine uptake among Black US residents.^56^ We recommend developing communication strategies prioritizing education over coercion and addressing the specific concerns Black women raised in this study. Engaging trusted community leaders, acknowledging historical medical exploitation,^26,34,57,58^ and leveraging personal stories to counter misinformation and build trust are essential steps toward reducing hesitancy. Providing transparent information about vaccine development, safety, and long-term effects, particularly addressing concerns about reproductive health, is crucial. Policymakers should consider these findings when developing strategies to increase vaccine uptake. This may include investing in community-based health initiatives and implementing policies to address broader health disparities contributing to mistrust in the government and health care system.

### Limitations

While this study provides valuable insights into the factors influencing COVID-19 vaccine hesitancy among Black women, it is important to acknowledge its limitations. As with all qualitative studies, our findings are not intended to be generalizable but to provide in-depth understanding of participants’ perspectives. The self-selection of participants may have attracted individuals with strong opinions, potentially missing perspectives from those less motivated to participate. The predominance of Southern participants (over 75%) limits the geographical diversity of our sample, potentially omitting unique perspectives from other regions and failing to fully account for differences in state-level policies such as mask mandates and vaccine distribution strategies, which may have informed participants’ experiences and vaccine hesitancy. We did not collect data on pregnancy status or intentions to conceive during our initial demographic data collection, which could have provided additional context for vaccine hesitancy. The homogeneity of our sample in terms of college education, religion, and geography limited our ability to draw clear distinctions based on these factors.

## Conclusions

This study provides insights into the complex factors contributing to COVID-19 vaccine hesitancy among Black women in the US. Study findings emphasize the need for a paradigm shift in how health care systems and public health initiatives engage with Black women, emphasizing the importance of addressing historical mistrust, creating culturally competent vaccine communication strategies that inform rather than coerce, and leveraging Black women’s voices and personal experiences. Health care practitioners can use these findings to improve communication with Black women patients, while public health officials can design more respectful outreach programs addressing specific concerns, which may increase vaccine confidence and uptake. Future research should examine a more geographically diverse sample to capture regional variations in vaccine hesitancy among Black women. Studies should explore the impact of pregnancy status and intentions to conceive on vaccine attitudes. Future work should also employ stratified sampling to better examine potential differences based on education, religion, and geography for a more comprehensive understanding of vaccine hesitancy across diverse subgroups of Black women.

## Notes

Supplement.
Data Sharing Statement
