# Supplementary material for: COVID-19 Vaccine Hesitancy Among Black Women in the US
Source: JAMA Netw Open. 2025 Jan 9;8(1):e2453511. doi: 10.1001/jamanetworkopen.2024.53511 (PMC11718551; doi:10.1001/jamanetworkopen.2024.53511)
Supplement: Supplement. — Data Sharing Statement [file jamanetwopen-e2453511-s001.pdf]

Data Sharing Statement

Slatton. COVID-19 Vaccine Hesitancy Among Black Women in the US. *JAMA Netw Open*. Published January 09, 2025. doi:10.1001/jamanetworkopen.2024.53511

Data
Data available: No

Additional Information
Explanation for why data not available: To protect participant privacy and maintain confidentiality consistent with the informed consent agreement and ethical guidelines for qualitative research, the raw qualitative data from this study will not be made available for public use.
